# 17p13.3 genomic rearrangement in a Chinese family with split-hand/foot malformation with long bone deficiency: report of a complicated duplication with marked variation in phenotype

**DOI:** 10.1186/s13023-018-0838-y

**Published:** 2018-07-03

**Authors:** Yuqi Shen, Nuo Si, Zhe Liu, Fang Liu, Xiaolu Meng, Ying Zhang, Xue Zhang

**Affiliations:** 1 McKusick-Zhang Center for Genetic Medicine, Institute of Basic Medical Sciences Chinese Academy of Medical Sciences, School of Basic Medicine Peking Union Medical College, No.5 Dong Dan San Tiao, Dongcheng District, Beijing, 100005 China; 2 Laboratory of Clinical Genetics, Peking Union Medical College Hospital, Chinese Academy of Medical Sciences & Peking Union Medical College, No.1 Shuaifuyuan, Wangfujing, Dongcheng District, Beijing, 100073 China; 3 Department of Obstetrics and Gynecology, Tianjin Medical University General Hospital, No.154, Anshan Road, Heping District, Tianjin, 300052 China

**Keywords:** Split hand/foot malformation with long bone deficiency, 17p13.3 genomic rearrangement, *BHLHA9*

## Abstract

**Background:**

Split hand/foot malformation (SHFM) is a genetically heterogeneous limb malformation with variable expressivity. SHFM with tibia or femur aplasia is called SHFM with long bone deficiency (SHFLD). 17p13.3 duplications containing *BHLHA9* are associated with SHFLD. Cases with variable SHFLD phenotype and different 17p13.3 duplicated regions are reported. The severity of long bone defect could not be simply explained by *BHLHA9* overdosage or 17p13.3 duplication.

**Methods:**

A four-generation Chinese SHFM family was recruited. Three family members have long bone defects, one male was severely affected with hypoplasia or aplasia in three of four limbs. Linkage analysis and direct sequencing of candidate genes were used to exclude six responsible genes/loci for isolated SHFM. Array comparative genomic hybridization (CGH) was performed to detect copy number variations on a genome-wide scale, and quantitative real-time polymerase chain reaction (qPCR) assays were designed to validate the identified copy number variation in the index and other family members.

**Results:**

No mutations were found in genes or loci linked to isolated SHFM. A ~ 966 kb duplication was identified in 17p13.3 by array CGH, in which *BHLHA9* surrounding region presented as triplication. The qPCR assays confirmed the indicated 17p13.3 duplication as well as *BHLHA9* triplication in all available affected family members and other two asymptomatic carriers. Given the incomplete penetrance in SHFLD, those two carriers were regarded as non-penetrant, which suggested that the genomic rearrangement was co-segregated with malformation in this family.

**Conclusions:**

The present study reports an additional SHFLD family case with 17p13.3 genomic rearrangement. To our knowledge, the 966 kb genomic rearrangement is larger in size than any previously reported SHFLD-associated 17p13.3 duplication, and the present family shows marked phenotypic variability with two asymptomatic carriers and one patient with an extremely severe phenotype. This rare case provides the opportunity to identify underlying genotype-phenotype correlations between SHFLD and 17p13.3 genomic rearrangement.

**Electronic supplementary material:**

The online version of this article (10.1186/s13023-018-0838-y) contains supplementary material, which is available to authorized users.

## Background

Split-hand/foot malformation, affecting 1 in 8500–25,000 newborns, is a developmental limb malformation characterized by median clefts of the hands and feet, syndactyly, and aplasia and/or hypoplasia of the phalanges, metacarpals, and metatarsals [1]. However, the phenotype varies greatly between families, within members of one family, and even among different extremities of a single individual, ranging from slight syndactyly and/or ectrodactyly to severe monodactyly. Currently, SHFM is categorized as either non-syndromic or syndromic, in terms of whether there are extra-limb manifestations. Non-syndromic SHFM includes isolated SHFM; and SHFM in association with other limb manifestations, such as tibia or femur aplasia, is called SHFM with long bone deficiency (SHFLD), and others involving fibula defects are designated as fibular agenesis with ectrodactyly (OMIM 113310).

Until now, six genes/loci (SHFM1–6) have been identified as responsible for isolated SHFM. These include deletions, duplications, or rearrangements on 7q21.2-q21.3, heterozygous/homozygous mutations in *DLX5* or heterozygous mutations in *DLX6* (SHFM1, OMIM 183600), heterozygous duplications on 10q24 (SHFM3, OMIM 246560), heterozygous mutations in *TP63* (SHFM4, OMIM 605289), heterozygous deletions on 2q31 (SHFM5, OMIM 606708) and biallelic mutations in *WNT10B* (SHFM6, OMIM 225300); in addition, SHFM2 has been mapped to Xq26.3 by linkage analysis. Causative genes in isolated SHFM are also responsible for some syndromic SHFM. For example, dominant *TP63* mutations can also result in ectrodactyly, ectodermal dysplasia, and cleft lip/palate syndrome 3 (EEC3, OMIM 604292).

SHFLD is genetically distinct from isolated SHFM. SHFLD is usually inherited in an autosomal dominant manner with variable expressivity and incomplete penetrance, and in some cases, autosomal recessive inheritance is presented, and digenic inheritance can also be possible. In general, SHFLD is associated with three different loci: 1q42.2-q43 for SHFLD1 (OMIM 119100); 6q14.1 for SHFLD2 (OMIM 610685); and 17p13.3 for SHFLD3 (OMIM 612576). The first two SHFLD susceptibility loci, 1q42.2-q43 and 6q14.1, were identified in the same large consanguineous family by genome-wide linkage analysis and multipoint parametric linkage analysis [2, 3], raising the possibility of digenic inheritance. For SHFLD3, 17p13.3 duplications have been identified in several cases and families, and the smallest duplication region only includes *BHLHA9* [4–10]. BHLHA9 plays a crucial role in limb development during apical ectodermal ridge (AER) formation, and its dosage has been implicated in SHFM and SHFLD [11]. SHFLD with 17p13.3 duplication is less than 50% penetrant and shows markedly variable expressivity. The correlation between a specific phenotype and the 17p13.3 duplications remain unclear. Although *BHLHA9* duplication is highly associated with long-bone deficiency, it is insufficient to explain the variable expressivity and reduced penetrance in SHFM and SHFLD.

Here, we present a genetic analysis of a Chinese family with SHFM and SHFLD. A ~ 966 kb genomic rearrangement in 17p13.3 is associated with the observed malformations in the family. Variable expressivity and incomplete penetrance are demonstrated in the family.

## Methods

### Subjects

A four-generation Chinese family with SHFM and SHFLD was recruited from the Department of Obstetrics and Gynecology of Tianjin Medical University General Hospital. Clinical data and digital photographs of limbs and digits were obtained from the patients and their referring doctor. Peripheral or cord blood were collected from 13 family members to perform further genetic analyses. Genomic DNA was extracted from blood leucocytes, according to standard procedures of the QIAamp DNA Blood Midi Kit (Qiagen, Valencia, CA, USA). Informed consent was obtained from all available individuals included in this study and the study was approved by the Ethical Review Board of Peking Union Medical College.

### Sanger sequencing of candidate genes

Mutations in all coding exons and intron-exon boundaries of candidate genes, including *TP63, DLX5, DLX6* and *BHLHA9,* were screened by Sanger sequencing. Primers used to amplify the target sequences were designed by Primer Premier 5 (Premier Biosoft International, Palo Alto, CA). Each polymerase chain reaction (PCR) was conducted in a 25 μl reaction mixture using LA Taq with GC Buffer I (Takara Bio., Dalian, PR China). The PCR products were directly sequenced in an ABI Prism 3730xl Automated Sequencer (Applied Biosystems, Foster City, CA, USA), and sequences were analyzed with CodonCode Aligner software (CodonCode Corp., Dedham, MA, USA). The primers are summarized in Additional file 1: Table S1.

### Linkage analysis for candidate loci

At least 4 short tandem repeat (STR) markers were selected within each candidate loci, including 7q21.2-q21 (SHFM1), Xq26.3 (SHFM2), and 10q24.3 (SHFM3). The STR markers were amplified by PCR and genotyped by polyacrylamide gel electrophoresis (PAGE). The primer sequences and amplification conditions are available upon request. Segregation status between genotypes and phenotypes were analyzed to determine the possibility of linkage to each locus.

### Microarray analysis

Array-CGH was performed on patient I1 using the Agilent Sureprint G3 Custom CGH microarray 2x400k (Agilent Technologies, Santa Clara, CA, USA) by Beijing Capitalbio Technology Corporation (Beijing, China). Genomic copy number changes at locus 17p13.3 in other family members (II1, II6, II8, III6, IV5, IV6) were further tested using a microarray with higher probe density (SurePrint G3 Human 1x1M; manufactured by Agilent Technologies, Santa Clara, CA, USA) by the Laboratory of Clinical Genetics of Peking Union Medical College Hospital. The experiment and data analysis were performed according to the manufacturer’s instructions. In brief, patient and control DNA were labeled and combined to hybridize to the 60-mer oligonucleotide-based microarray. The resulting fluorescent signals were automatically scanned by the Agilent SureScan Microarray Scanner. Agilent CytoGenomics software was then used to extract and translate the signal into log ratios for further analysis of copy number changes.

### Quantitative real-time PCR assay

A quantitative real time PCR (qPCR) assay was used to test for duplication of the 10q24.3 and 17p13.3 loci in all available DNA samples. The qPCR was performed in a total volume of 20 μl with each tube containing 2× SYBR Premix *Ex Taq* GC (Takara Bio., Dalian, PR China), 50 ng genomic DNA, and 400 nM each primer, with four replicates per sample. Reactions were performed in a Rotor-Gene 6000 instrument (Qiagen, Hilden, Germany) under the following conditions: 95 °C for 10 min and 40 cycles of 95 °C 10s / 60 °C 15 s / 72 °C 20s. Data were analyzed by Rotor Gene Q series software (Qiagen, Hilden, Germany). The relative copy number (RCN) of the target sequence was calculated by the relative threshold cycle method (2^-ΔΔCt^) where ΔCt = (mean Ct_Target_)-(mean Ct_Reference_) and ΔΔCt = ΔCt_patient_-ΔCt_control_. A RCN of approximately 1.5 indicated a heterozygous duplication.

## Results

### Clinical description

**Fig. 1:**
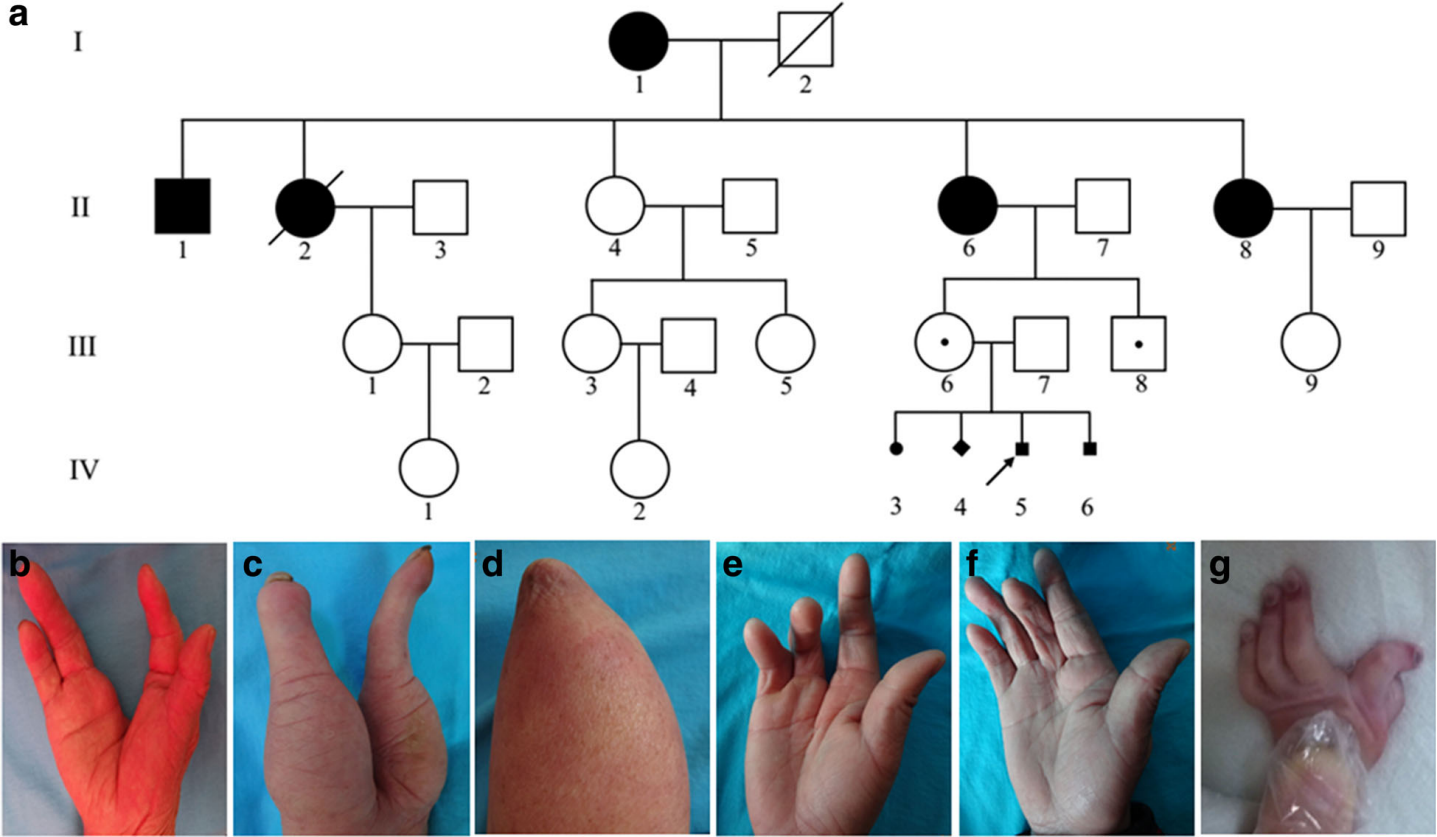
Pedigree and limb defects of affected subjects. **a** Pedigree showing dominant inheritance with reduced penetrance. **b** Ectrodactyly with split hand of patient I1. **c-d** Ectrodactyly with split left hand of II1 (**c**). The hypoplastic right forearm with no hand, ulna and radius of II1 (**d**). Patient II1 also affected with symmetric hypoplastic femora and absence of bilateral lower legs. **e** Unilateral ectrodactyly with split hand of II6. **f**. Unilateral clinodactyly in middle digit of II8. **g** Abnormal left split hand of the aborted fetus IV6

Multiple family members, including five individuals and four fetuses, presented limb malformation with extreme phenotypic diversity (Table 1). The proband (IV5) was evaluated for unilateral syndactyly displayed in a mid-trimester ultrasound before his mother (III6) obtained an abortion. Patient III6 is phenotypically normal, and has obtained four abortions, all because of limb malformations detected in the fetus by ultrasound, occurring twice before, and once after, patient IV5. There were one female and two male fetuses, and one whose gender is not available. The first female fetus (IV3) was observed with bilateral ectrodactyly, and syndactyly with a central cleft, and bilateral leg flexion deformity. Fetus IV4, whose gender is not available, was observed to have bilateral ectrodactyly and metacarpal reduction defect in the upper limbs, and bilateral tibia or fibula reduction defect and leg flexion deformity in the lower limbs. Fetus IV6 only presented with a malformation in his left hand, with a wide cleft between the second and third fingers (Fig. 1g). Dating back two generations before III6, there are five patients in the family. I1 had bilateral ectrodactyly with a central cleft (Fig. 1b), and four of her five children presented with limb malformations including II6, the mother of III6. The clinical manifestation of II1 was the most severe, with his left hand only contained two digits with a central cleft (Fig. 1c), the right forearm with hypoplastic ulna and radius, and no developed hand (Fig. 1d), along with legs that presented with symmetric hypoplastic femora and absence of the lower legs. Patients II2 and II6 presented unilateral ectrodactyly with a central cleft, and II6 also showed clinodactyly in the third and fourth digits (Fig. 1e). II8 showed syndactyly at birth and clinodactyly in the third digit after corrective surgery (Fig. 1f). The other individuals in this family had no significant anomalies in any limb or digit. The malformation manifested primarily in the hands of the family with variable expressivity, notably, three individuals had long bone aplasia. Obligate carrier (III6) demonstrated that the malformation could be transmitted in an autosomal dominant, or X-linked mode with incomplete penetrance. No other obvious health issues were present in the family; cognitive ability, behavior, and development were normal in all individuals.

**Table 1:** Limb features of family members

Individual	Gender	Upper limbs		Lower limbs		Phenotype	17p13.3 genomic rearrangement
		Left	Right	Left	Right		
I1	F	Ectrodactyly with a central cleft	Ectrodactyly with a central cleft	Normal	Normal	SHFM	Y
II1	M	Ectrodactyly with a central cleft	Forearm reduction defect	Femora hypoplasia, lower leg reduction defect	Femora hypoplasia, lower leg reduction defect	SHFLD	Y
II2	F	Unilateral ectrodactyly with a central cleft		Normal	Normal	SHFM	NT
II4	F	Normal	Normal	Normal	Normal	Normal	N
II6	F	Normal	Ectrodactyly with a central cleft, clinodactyly in the third and fourth digits	Normal	Normal	SHFM	Y
II8	F	Normal	Syndactyly	Normal	Normal	SHFM	Y
III6	F	Normal	Normal	Normal	Normal	Carrier	Y
III8	M	Normal	Normal	Normal	Normal	Carrier	Y
III9	F	Normal	Normal	Normal	Normal	Normal	N
IV3	F	Ectrodactyly and syndactyly with a central cleft	Ectrodactyly and syndactyly with a central cleft	Leg flexion deformity	Leg flexion deformity	SHFLD?	NT
IV4	NA	Ectrodactyly, metacarpale reduction defect	Ectrodactyly, metacarpale reduction defect	Tibia or fibula reduction defect, leg flexion deformity	Tibia or fibula reduction defect, leg flexion deformity	SHFLD	NT
IV5	M	Syndactyly	Normal	Normal	Normal	SHFM	Y
IV6	M	Split hand between index and other three fingers	Normal	Normal	Normal	SHFM	Y

F, female; M, male; NA, not available; Y, yes; N, no; NT, not tested

### Genetic analysis

#### Mutation detection in known SHFM causative genes / loci

Mutation screening was performed in genes and at loci that had been previously linked to SHFM. No potential pathogenic mutation was identified in the coding region of *DLX5, DLX6* (SHFM1) or *TP63* (SHFM4) by direct sequencing. Copy number changes were not detected at the SHFM3-associated locus 10q24.3 by qPCR assay. There were shared alleles among patients in the family at 7q21.2-q21 (SHFM1) and Xq26.3 (SHFM2), however, considering the relatively small number of patients and the presence of incomplete penetrance, the linkage to these two loci cannot be confirmed. SHFM5 usually presents growth retardation and craniofacial anomaly, and SHFM6 is inherited in an autosomal recessive manner. Since no featured clinical presentation was observed in these patients, and the inheritance was obviously not consistent with an autosomal recessive mode, the SHFM5 and SHFM6 types were excluded.

#### Detection of whole genome copy number variation

**Fig. 2:**
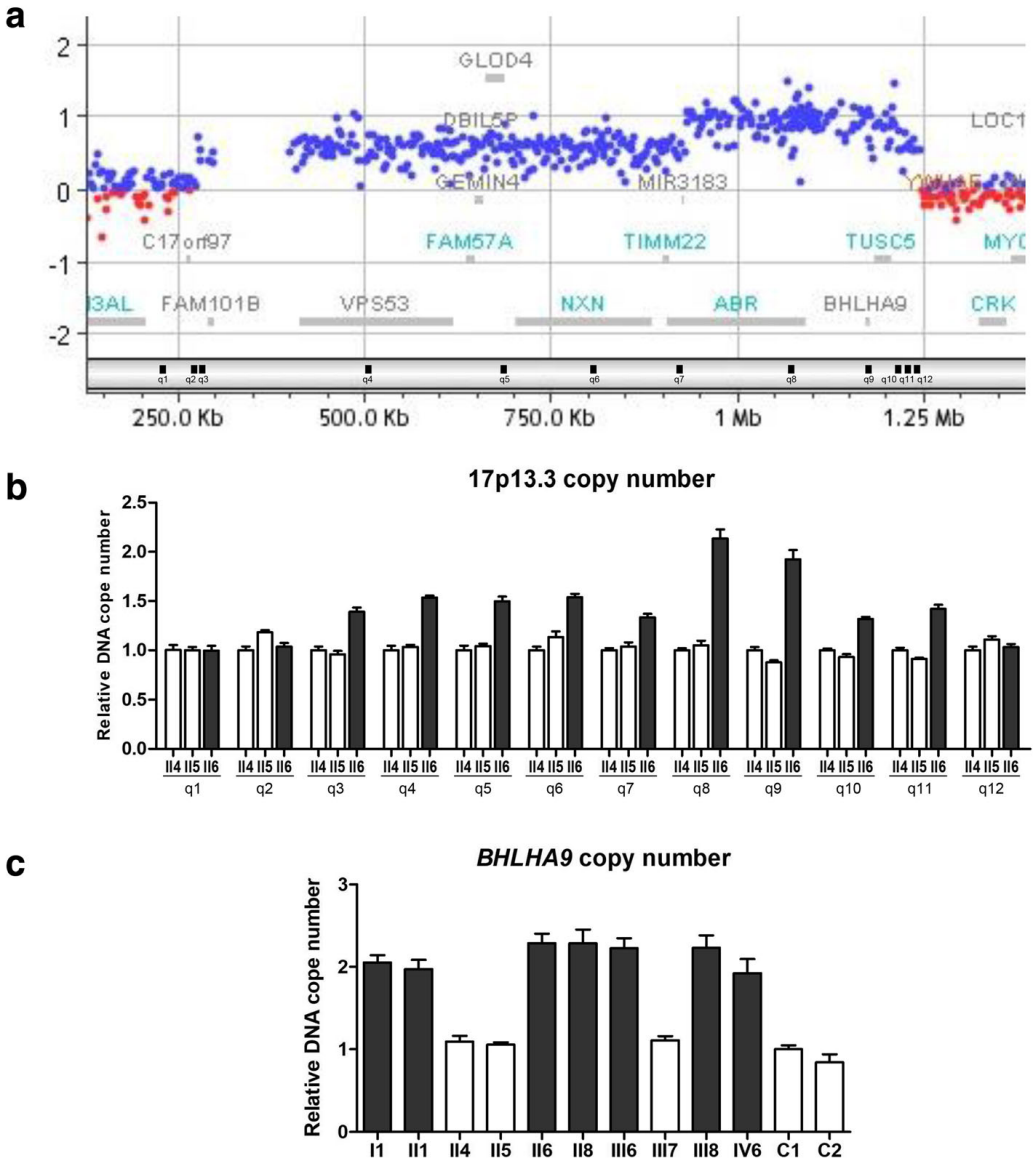
Identification and validation of 17p13.3 genomic rearrangement. **a** Array CGH results (GRCh37/hg19 build) showed copy number gain at the chromosome 17p13.3 locus. A large duplication was identified here and part of this region encompassing *ABR1* and *BHLHA9* manifested as triplication. The blue and the red dots denote amplification and deletion, respectively. The log ratio (Y axis) demonstrated by the blue dots indicated the presence of increased copies of this region. Ratios around + 0.5 and around + 1.0 indicate the presence of three and four copies of its corresponding region, respectively. **b** DNA copy numbers of 17p13.3 genomic rearrangement region detected by qPCR assays. Primers locations are indicated at the bottom of **a** (black bars). **c** DNA copy number of *BHLHA9* detected by qPCR assays. All the normal subjects except III6 and III8 in this family show normal copy number (white bars). All the affected individuals show increased copy numbers (black bars). C1 and C2 are two normal non-familial individuals, which represent a male and a female respectively

The copy number variation in the family was examined at a genome-wide scale. High resolution array CGH analysis in patient I1 revealed a genomic rearrangement at 17p13.3, presenting a gross duplication, in which *BHLHA9* surrounding region presented as triplication (hg19) (Fig. 2a). Further analyses in confirmed patients (II1, II6, II8, IV5, IV6), and an obligate carrier (III6), also revealed the similar results. The size of the genomic rearrangement as detected by array CGH ranged from 793 kb to 972 kb: 972 kb in IV5 (chr17: 271755–1,244,126); 967 kb in I1 (chr17: 277049–1,244,126) and III6 (chr17: 277249–1,244,126); 804 kb (chr17: 410709–1,215,190) in II1 and II6; 793 kb (chr17: 418332–1,211,150) in II8; and 833 kb (chr17: 410709–1,244,126) in IV6. Thus, the 17p13.3 genomic rearrangement was observed in all available affected family members and co-segregated with malformation in the family. However, III6 also carried this genomic rearrangement while showing no limb or digit anomalies, confirming the existence of nonpenetrance at this locus. No additional aberrations were observed at other SHFM/SHFLD loci in the patients.

#### qPCR at 17p13.3 and sanger sequencing in *BHLHA9*

qPCR primers were designed within the 17p13.3 genomic rearrangement region identified by array CGH, to confirm the gross genomic rearrangement and narrow down the boundaries. This result showed that the duplication was ~ 966 kb in size on chr17:276288–1,242,170 and one region within it encompassing *ABR1* and *BHLHA9* showed triplication (Fig. 2b). The previously reported minimally duplicated region at 17p13.3 that has been associated with SHFLD contains only one gene, *BHLHA9*, which was also present in the identified genomic rearrangement region in the present study. Therefore, a qPCR assay was performed on *BHLHA9* to confirm the copy number gain in all available family members and two non-familial members. All affected individuals showed increased two copy numbers compared to normal subjects (Fig. 2c). Copy number gain was also observed in two phenotypically normal members, III6 and III8, which suggested these two were asymptomatic carriers and demonstrated the existence of nonpenetrance in the family. The sequences of these qPCR primers are summarized in Additional file 1: Table S1. In addition, based on the potential pathogenesis of gain-of-function mutations of *BHLHA9* in limb malformations, the *BHLHA9* sequence was also examined by Sanger sequencing in the proband, and no potential pathogenic variant was found*.*

## Discussion

In the present family, a total of nine members were affected with limb malformation, including four fetuses. Most patients manifested as SHFM with unilateral or bilateral hand(s) involved, while lower limb involvement was present in one third of the patients. One male patient (II1) presented a severe SHFLD phenotype with long-bone deficiency in three of four limbs, which included a hypoplastic right forearm, and bilateral absence of lower legs, in addition to a split left hand. According to previous reports, only 43% of SHFM patients with 17p13.3 duplication are affected with long bone deficiency, with tibia involved in most cases, but also with radial hypoplasia, femur hypoplasia, and a reduction defect of upper and/or lower limbs [9]. To our knowledge, patient II1 of this study is the most severe SHFLD case in all reported 17p13.3 genomic rearrangement carriers. The most severe phenotypes previously reported were reduction defects of the right upper and lower limbs with a split left hand in a fetus [9], while all four limbs of patient II1 herein were involved, of which three of four limbs were seriously dysplasic or aplasic. Whether there is a sex bias in the incidence and severity of SHFLD is still controversial [9, 10, 12]. In Caucasian patients, a clear sex bias has been observed, with more males affected than females [12], while no significant sex difference has been observed in Japanese populations [10]. In this study, one severe SHFLD patient is observed out of four males, one suspicious case with bilateral leg flexion deformity out of six females, and one SHFLD case with unknown gender in the whole family. Two asymptomatic carriers, one male and one female, were also observed. Limited by the small sample size, the relationship between gender and the severity and incidence of long-bone deficiency is speculated, but not confirmed, in this family.

A ~ 966 kb genomic rearrangement was identified at 17p13.3 in a four-generation Chinese family with nine individuals affected with SHFM or SHFLD. Within this large region, there was one part containing *ABR1* and *BHLHA9* presenting triplication, which showed a complicated structural variation in this region. The phenotypes associated with the 17p13.3 duplications included neurological dysfunction, cleft lip/palate, SHFLD, and connective tissue phenotypes [6]. To our knowledge, *YWHAE* duplication is responsible for the neurological dysfunction phenotype [13], and a duplication disrupting *ABR* appears to result in cleft lip/palate [6], while the SHFLD phenotype has been associated with duplication of *BHLHA9* [5, 12]*.* The identified duplication in our study does not contain *YWHAE*, and it encompasses *ABR*, along with *BHLHA9.* Accordingly, patients in this family were only affected with limb malformations, but no neurological disorders or cleft lip/palate were observed. These observations are in accordance with the genotype-phenotype correlations summarized by Curry et al. [6]. Increased *BHLHA9* copy numbers have been reported to be highly correlated with the long bone deficiency phenotype. According to Nagata et al., the incidence of long bone defect is significantly higher in families with *BHLHA9* triplicates than with duplicates [10]. In this study, the increased copy number of 17p13.3 containing *BHLHA9* was confirmed by qPCR assay, and it was considered to be responsible for the phenotypes observed in this Chinese family. In addition to previous studies in Caucasian and Japanese patients, the same genomic variations were observed in Chinese cases for the first time, indicating that increased *BHLHA9* copy number is associated with SHFLD in different populations.

**Fig. 3:**
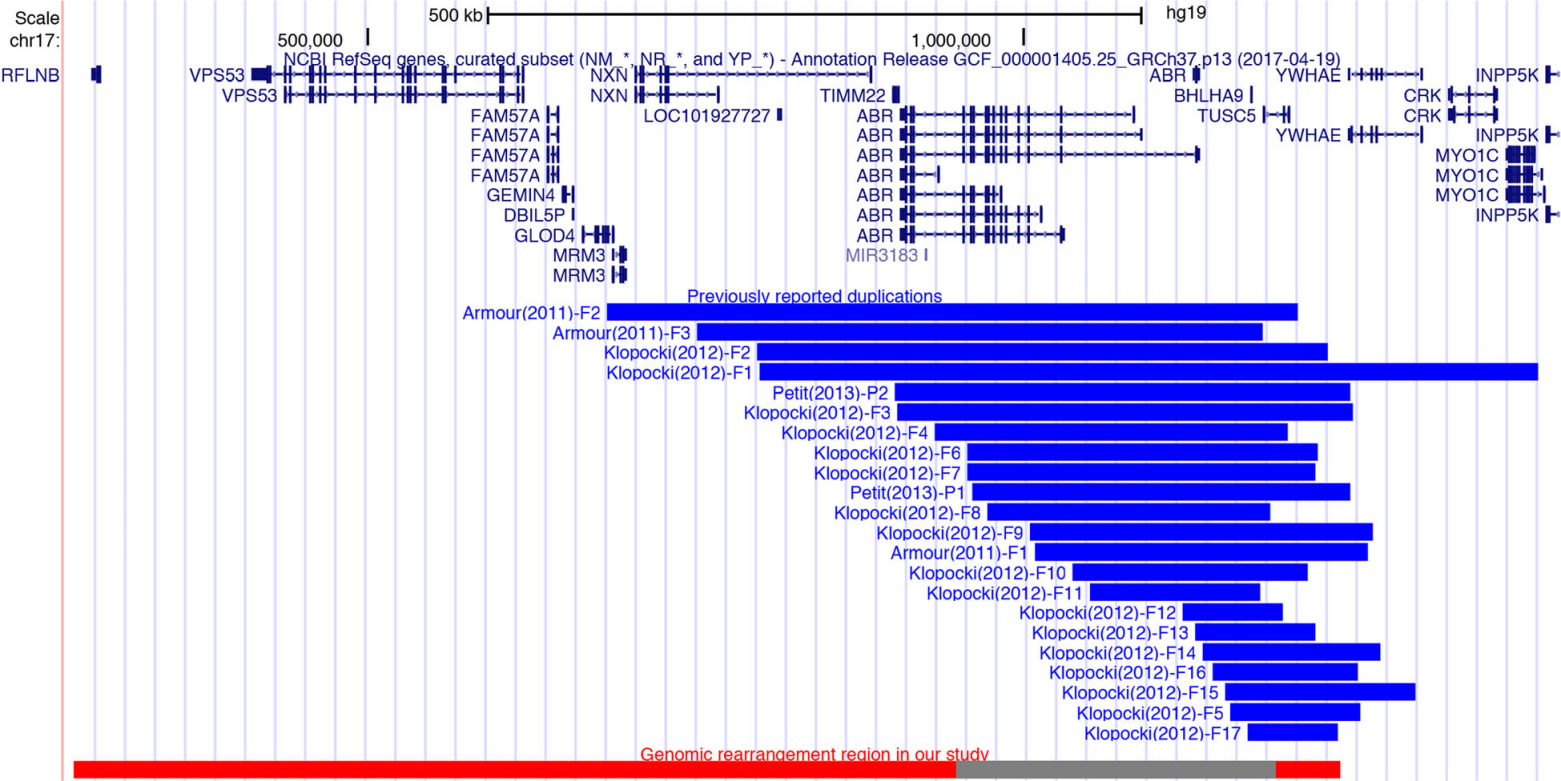
Schematic overview of the 17p13.3 duplications previously reported and identified in our study (hg19). All the twenty-two blue blocks denote the previously reported duplicated regions. The lower red block denotes the identified duplication and the grey block denotes the identified triplication in our study. The complicated duplication found in our study contained a wider telocentric region than in previously reported 17p13.3 duplications

The ~ 966 kb genomic rearrangement identified in this family is the largest SHFLD associated 17p13.3 structural variation identified to date, and the long bone deficiency phenotype in one family member is extremely severe. Previously, the largest duplication identified in SHFLD patients was 594 kb, and the minimum critical region only contained a single *BHLHA9* [12]*.* Compared with the previously identified 594 kb duplication, the complicated duplication described here encompassed five additional genes, *RFLNB, VPS53, FAM57A, GEMIN4*, and *DBIL5P* (Fig. 3a). No involvement in limb development of *VPS53, FAM57A, GEMIN4*, and *DBIL5P* has been reported up to now, thus the severe phenotype in this family could not be explained simply by the duplication of these four genes. Although *RFLNB* has been implicated in regulating differentiation and proliferation of chondrocytes, the *Rflnb* knock-out mice are phenotypically normal [14], thus the role of *RFLNB* in limb malformation remains unknown. It is currently believed that overexpression of *BHLHA9* is the major associated factor of SHFLD. *BHLHA9* plays a significant role in limb development. It is expressed in the distal limb bud mesenchyme underlying the AER in mouse embryos [12]. *BHLHA9* loss-of-function mutation results in limb malformation in human and animal models. Knocking down *Bhlha9* in zebrafish leads to truncated pectoral fins [12], and *Bhlha9-*knockout mice show syndactyly [11]. Moreover, loss-of-function mutations in the *BHLHA9* DNA-binding domain can cause autosomal-recessive inherited mesoaxial synostotic syndactyly (MSSD, OMIM 609432) [15]. No mutation was observed in the present family by sequencing the entire *BHLHA9* coding region, and the inheritance pattern shown here was autosomal dominant, thus the severe phenotype was not caused by *BHLHA9* loss of function. The BHLHA9 dosage is highly correlated to the long bone defect phenotype [10]. We identified a *BHLHA9*-containing triplication, while the phenotypes in the present family were more severe than a *BHLHA9*-containing triplication in Japanese populations [10]. Therefore, the severe phenotype in this family could not be simply explained by the increased copy number of *BHLHA9* as well. Gene expression dosage can be regulated by multiple factors, such as a long-range cis-acting regulatory element. Disruption of long-range cis-acting regulatory elements has been demonstrated to be responsible for several limb malformations. For example, microduplications and mutations in the ZRS enhancer, a well-known long-range cis-element of Sonic hedgehog (*SHH*), is associated with triphalangeal thumb-polysyndactyly syndrome and preaxial polydactyly [16]. It is noteworthy that the ZRS enhancer is about 980 kb upstream of *SHH*, which is similar to the genomic rearrangement size found in this study. Therefore, it is possible that the genomic rearrangement region identified here contains a long-range cis-acting regulatory element of *BHLHA9*. Since the smaller duplication only includes *BHLHA9* and nearby regulatory elements, it is assumed that gene expression is influenced mainly by the increased copy number of *BHLHA9* and the nearby promoter and enhancers. However, since the large genomic rearrangement includes both long-range regulatory elements and *BHLHA9*, gene expression is also influenced by enhanced long-range regulation, and, as a result, the expression level is much greater and the phenotype is more severe. The long-range regulatory element may not be included in the previously reported duplicated regions, which could explain why Petit et al. and Klopocki et al. did not observe a correlation between the duplication size and phenotype severity [7, 12]. Further evidence should be provided to support this assumption, including additional severe SHFLD patients with large genomic rearrangements along with the direct identification of a long-range cis-acting regulatory element of the *BHLHA9* gene. In addition, incomplete penetrance and phenotypic variability are often observed in SHFLD patients, such as observed in the family in this study. Particularly, a patient-to-carrier-to-patient transmission pattern was observed in the present family, suggesting the involvement of additional factors from the complicated genetic and environmental backgrounds. The highly variable expressivity and incomplete penetrance, particularly the asymptomatic carriers, imply that an individual with a 17p13.3 genomic rearrangement does not certainly present a malformation phenotype, which perplex the prenatal diagnosis and genetic counseling in the present family.

## Conclusions

In summary, this study reported an additional SHFLD family case with 17p13.3 genomic rearrangement. To our knowledge, the 966 kb genomic rearrangement is larger in size than previously reported SHFLD-associated 17p13.3 duplications, and the present family shows marked variation in phenotype, with two asymptomatic carriers, and one patient with an extremely severe phenotype. The rare case provides us an opportunity to observe the underlying genotype-phenotype correlation between SHFLD and a 17p13.3 genomic rearrangement containing *BHLHA9*.

## Additional file

Additional file 1: **Table S1.** Primers used in this study. (DOCX 27 kb)

## Abbreviations

AER: Apical ectodermal ridge
EEC3: Ectodermal dysplasia and cleft lip/palate syndrome 3
MSSD: Mesoaxial synostotic syndactyly
PAGE: Polyacrylamide gel electrophoresis
PCR: Polymerase chain reaction
qPCR: Quantitative real time PCR
SHFLD: SHFM with long bone deficiency
SHFM: Split hand/foot malformation
SHH: Sonic hedgehog.
STR: Short tandem repeat
