# Supplementary material for: 17p13.3 genomic rearrangement in a Chinese family with split-hand/foot malformation with long bone deficiency: report of a complicated duplication with marked variation in phenotype
Source: Orphanet J Rare Dis. 2018 Jul 3;13:106. doi: 10.1186/s13023-018-0838-y (PMC6029155; doi:10.1186/s13023-018-0838-y)
Supplement: Supplementary file 1 — Table S1. Primers used in this study. (DOCX 27 kb) [file 13023_2018_838_MOESM1_ESM.docx]

**Table S1. Primers used in this study.**

| Genes/Loci | Exons / Loci (hg 19) | Sequence from 5′ to 3′ | Description |
| --- | --- | --- | --- |
| *TP63* | 1 | F: GTCTGATAGCATTTGACCCTATTG | PCR and Sanger sequencing |
|  |  | R: GACACATTCATAATACACAAGGCAC |  |
|  | 2 | F: TGTGCAACTTGTAACATGTGCTAC |  |
|  |  | R: GCAATATTTTGACCACCCACAT |  |
|  | 3 | F: TTGTTGTTAACAACAGCATGAG |  |
|  |  | R: GAAAAGACAGGTTTAACAGAGC |  |
|  | 4 | F: TTGGCAAAATCCTGGAGCC |  |
|  |  | R: GACCGAGAACCGCAAATACG |  |
|  | 5 | F: CCGACGTGAGGTCCATCTCT |  |
|  |  | R: GGAGAAATCCCTGGGACGTG |  |
|  | 6 | F: GTTGGTTCTCTCCTTCCTTTC |  |
|  |  | R: GCCCACAGAATCTTGACCTTC |  |
|  | 7 | F: CACCAACATCCTGTTCATGC |  |
|  |  | R: CATGTTACCAAGTTGATTCAGTGC |  |
|  | 8 | F: GGGAAGAACTGAGAAGGAACAAC |  |
|  |  | R: CAGCCACGATTTCACTTTGCC |  |
|  | 9 | F: GTAGATCTTCAGGGGACTTTC |  |
|  |  | R: GACGTCCCACTTGCTGCTGAAG |  |
|  | 10 | F: GCTTTAGAAGTGTTCCCAGG |  |
|  |  | R: ACACCTCCTTTCCCATTGTC |  |
|  | 11 | F: TGAGGATTGACCACACTTCTAAC |  |
|  |  | R: CATCAATCACCCTATTGCTGATC |  |
|  | 12 | F: GTGTTGGAAGGGAGCCAGG |  |
|  |  | R: GTGCCTTTGAGCAGTTGGG |  |
|  | 13 | F: ACCATTATTTCCATGTTTGTC |  |
|  |  | R: GCTTCCCCATCACAGAGTC |  |
|  | 14 | F: CTGGTAGTTTAGGCCCTTGAT |  |
|  |  | R: GGACTATAACAGTATCCGCCC |  |
|  | 15 | F: CTTATCTCGCCAATGCAGTTGG |  |
|  |  | R: AACTACAAGGCGGTTGTCATCAG |  |
|  | 16 | F: GGATCAATAGATTCAGATCAAT |  |
|  |  | R: AAGATTAAGCAGGAGTGCTT |  |
| *DLX5* | 1 | F: CGAGTTAGGGTGTTACTGTTGCT | PCR and Sanger sequencing |
|  |  | R: CCAGGAGCTGCTTGATTCTT |  |
|  | 2 | F: AGAAGCAACGACCGCAAG |  |
|  |  | R: CCAGACCGCTGATGAATACC |  |
|  | 3 | F: GGAGTAGGACGGGAGGTTG |  |
|  |  | R: GGGTTATTGAGGGATTTCTGTTG |  |
| *DLX6* | 1 | F: TGCAAGGATCCAAAGAGCTAAG | PCR and Sanger sequencing |
|  |  | R: GAGAGCGCAGTGTGGACTAACT |  |
|  | 2 | F: GGCTTTGGGGAGACTCGTTG |  |
|  |  | R: TCCTTCCATCCTTGGTTGACT |  |
|  | 3 | F: CTTGGCTTGTAGGCGTTGG |  |
|  |  | R: TTTCAGGAGTGCTGGCATAGA |  |
| *BHLHA9* | 1 | F: GAAGAATGTGGAACCCAGACAC | PCR and Sanger sequencing |
|  |  | R: CCTGAGCCTTTCTGGCAACT |  |
| *BHLHA9* | 1 / chr17:1174086-1174179 | F: CGCATCCTAGACTACAACGAGG | Determining the DNA copy number or expression by qPCR |
|  |  | R: GCAGCGTGGCGATCTTG |  |
| q1 | chr17:254698-254872 | F: CCAGCCTTTCCAAGCACC | Confirming the genomic rearrangement by qPCR |
|  |  | R: TGTGCGGTATCTGCCTCTT |  |
| q4 | chr17:500265-500400 | F: CTTTCTGTTATGCTTTCAGTGTCC | Confirming the genomic rearrangement by qPCR |
|  |  | R: CTGAGAAGAGAGAGGGAACCTG |  |
| q5 | chr17:700953-701037 | F: ATGCCAACACAGCACTCG | Confirming the genomic rearrangement by qPCR |
|  |  | R: GCTCTTGCTTTTCGCCC |  |
| q6 | chr17:807596-807734 | F: GAAGGACAAAAGGAAAGGACG | Confirming the genomic rearrangement by qPCR |
|  |  | R: TTGGGTCAAAGTGGAGGG |  |
| q7 | chr17:912936-913058 | F: CAGGAAGAGGAAGGTGATGAG | Confirming the genomic rearrangement by qPCR |
|  |  | R: AGCCAAACGCCACACTG |  |
| q8 | chr17:1213050-1213185 | F: AACCCATCTAACCACGCAC | Confirming the genomic rearrangement by qPCR |
|  |  | R: GTCTCCCACCCTGAGCATA |  |
| q9 | chr17:1175954-1176082 | F: AAAGTGCCTCTGCTGTCTGC | Confirming the genomic rearrangement by qPCR |
|  |  | R: GAGTCAAAGCCTTCCCGTG |  |
| q10 | chr17:1235100-1235216 | F: CAAACAACAGGGAGCTGCAC | Confirming the genomic rearrangement by qPCR |
|  |  | R: CCTGCCTCTCATGTAACCCC |  |
| Dup-L-q3 | [chr17:273109-273293](https://genome.ucsc.edu/cgi-bin/hgTracks?hgsid=642919139_twtxvGjgeFnqm3ZDjPTE6Elmqflb&db=hg19&position=chr17:273109-273293&hgPcrResult=pack) | F: CAAACCAGACCTACGGTATCAG | Determining the genomic rearrangement boundaries by qPCR. / No dup. |
|  |  | R: TCTATCTTCCAAGGCTATTCATCC |  |
| Dup-L-q4 | [chr17:274454-274592](https://genome.ucsc.edu/cgi-bin/hgTracks?hgsid=643435867_hIu1TYHtaCpkgMPl59bOhovtgoic&db=hg19&position=chr17:274454-274592&hgPcrResult=pack) | F: GAGAGAGCCTCATCTGTTTGG | Determining the genomic rearrangement boundaries by qPCR. / No dup. |
|  |  | R: GTGATCCTCCCACATCAGC |  |
| Dup-L-q5  (q2) | chr17:275753-275910 | F: CCCAGGTTCAAGTGATTCTCC | Determining the genomic rearrangement boundaries by qPCR. / No dup. |
|  |  | R: CAGATCACGAGGTCAGGAGTTC |  |
| Dup-L-q6  (q3) | chr17:276288-276371 | F: CAGGCATTCTGGCTCAAAG | Determining the genomic rearrangement boundaries by qPCR. / Dup. |
|  |  | R: CTTAGGAAAGGCAAAGAGGGC |  |
| Dup-L-q2 | [chr17:277378-277545](https://genome.ucsc.edu/cgi-bin/hgTracks?hgsid=640394951_E5jWesjJyC9vNHdl4WGfKaGyXMNf&db=hg19&position=chr17:277378-277545&hgPcrResult=pack) | F: AGCCCCTTCACGCTCTAAC | Determining the genomic rearrangement boundaries by qPCR. / Dup. |
|  |  | R: AAATCATTCGTATTCTCCCCAG |  |
| Dup-L-q1 | [chr17:290158-290267](https://genome.ucsc.edu/cgi-bin/hgTracks?hgsid=638476569_P6iEzn4I3b0iCSotysnKVmWsReAN&db=hg19&position=chr17:290158-290267&hgPcrResult=pack) | F: CCTCTGACCCACCCTGAAG | Determining the genomic rearrangement boundaries by qPCR. / Dup. |
|  |  | R: GAGCCAGTCGTAGTTCTTTGTAATC |  |
| Dup-R-q2 | [chr17:1238926-1239090](https://genome.ucsc.edu/cgi-bin/hgTracks?hgsid=643435867_hIu1TYHtaCpkgMPl59bOhovtgoic&db=hg19&position=chr17:1238926-1239090&hgPcrResult=pack) | F: AGACCACCTTACTCCTACAAGAATG | Determining the genomic rearrangement boundaries by qPCR. / Dup. |
|  |  | R: GACCTCACCTCAAGTGACCC |  |
| Dup-R-q3 | chr17:1238926-1239090 | F: CAAGTGGGCAAGATAAACGC | Determining the genomic rearrangement boundaries by qPCR. / Dup. |
|  |  | R: CGTATTAGTTCTAGTGGGCTGCTT |  |
| Dup-R-q4 | [chr17:1240783-1240894](https://genome.ucsc.edu/cgi-bin/hgTracks?hgsid=647999689_hnxVP8LP6pqnveXH4QoRtdfztagV&db=hg19&position=chr17:1240783-1240894&hgPcrResult=pack) | F: TTCCGCCATTTATCCACAAG | Determining the genomic rearrangement boundaries by qPCR. / Dup. |
|  |  | R: GGACATTTGGGTGTTTCCAG |  |
| Dup-R-q5  (q11) | [chr17:1242019-1242170](https://genome.ucsc.edu/cgi-bin/hgTracks?hgsid=643623743_FaN92xQzcYUtOiNXJei2lUNSCa9m&db=hg19&position=chr17:1242019-1242170&hgPcrResult=pack) | F: AAAATTAAGGGCTTCTGTTCCTC | Determining the genomic rearrangement boundaries by qPCR. / Dup. |
|  |  | R: TTTTCTGTTGGGATGTCGTTT |  |
| Dup-R-q6  (q12) | chr17:1242720-1242794 | F: CTGAGGCAGGAGAATCACTTC | Determining the genomic rearrangement boundaries by qPCR. / No dup. |
|  |  | R: GGCTGGAGTGTAGTGGCG |  |
| Dup-R-q1 | [chr17:1243863-1243961](https://genome.ucsc.edu/cgi-bin/hgTracks?hgsid=643172795_2FXMvpmu95wegS54A2WEyYD43x4e&db=hg19&position=chr17:1243863-1243961&hgPcrResult=pack) | F: TGGGCGATAGAGCAAGACC | Determining the genomic rearrangement boundaries by qPCR. / No dup. |
|  |  | R: ACTGTGCTGGACTGGAGGTAG |  |
